# Comprehensive Analysis of the Genetic and Epigenetic Mechanisms of Osteoporosis and Bone Mineral Density

**DOI:** 10.3389/fcell.2020.00194

**Published:** 2020-03-25

**Authors:** Hui Dong, Wenyang Zhou, Pingping Wang, Enjun Zuo, Xiaoxia Ying, Songling Chai, Tao Fei, Laidi Jin, Chen Chen, Guowu Ma, Huiying Liu

**Affiliations:** ^1^Department of Oral Prosthodontics, School of Stomatology, Dalian Medical University, Dalian, China; ^2^Department of Stomatology, The First Affiliated Hospital of Dalian Medical University, Dalian, China; ^3^School of Life Science and Technology, Harbin Institute of Technology, Harbin, China

**Keywords:** genome-wide association study, osteoporosis, bone mineral density, summary data-based Mendelian randomization, functional element enrichment analysis

## Abstract

Osteoporosis is a skeletal disorder characterized by a systemic impairment of bone mineral density (BMD). Genome-wide association studies (GWAS) have identified hundreds of susceptibility loci for osteoporosis and BMD. However, the vast majority of susceptibility loci are located in non-coding regions of the genome and provide limited information about the genetic mechanisms of osteoporosis. Herein we performed a comprehensive functional analysis to investigate the genetic and epigenetic mechanisms of osteoporosis and BMD. BMD and osteoporosis are found to share many common susceptibility loci, and the corresponding susceptibility genes are significantly enriched in bone-related biological pathways. The regulatory element enrichment analysis indicated that BMD and osteoporosis susceptibility loci are significantly enriched in 5′UTR and DNase I hypersensitive sites (DHSs) of peripheral blood immune cells. By integrating GWAS and expression Quantitative Trait Locus (eQTL) data, we found that 15 protein-coding genes are regulated by the osteoporosis and BMD susceptibility loci. Our analysis provides new clues for a better understanding of the pathogenic mechanisms and offers potential therapeutic targets for osteoporosis.

## Introduction

Osteoporosis is a systemic skeletal disease characterized by a significant decrease in BMD and microarchitectural deterioration of bone tissue (Rachner et al., 2011). The decline in bone mass and prevalence of osteoporosis increase with age, especially in postmenopausal women (Cummings and Melton, 2002). Researchers estimate there are more than 200 million individuals with osteoporosis worldwide, and the fracture risk of patients with osteoporosis is as high as 40% (Rachner et al., 2011; Al-Barghouthi and Farber, 2019). What’s worse, the number of patients with osteoporosis is expected to steadily increase in the near future, as the effects of an aging global population (Aggarwal and Masuda, 2018).

Osteoporosis, a typical of complex polygenic disease, is considered to be the consequence of the genetic interaction of multiple gene mutations (Saad, 2019). Previous studies based on twin and family data have estimated that both the osteoporosis and BMD show high heritability (*h*^2^ = 0.5–0.8) (Ralston and Uitterlinden, 2010; Al-Barghouthi and Farber, 2019). Clinically, BMD is a strong relevant marker of osteoporosis, as well as a key indicator for its diagnosis and treatment (Kemp et al., 2017). Therefore, a comprehensive understanding of the genetic factors underlying both osteoporosis and BMD is highly necessary to develop effective therapies for osteoporosis.

As early as in 1994, a candidate gene study found that several common allelic variants in vitamin D receptor gene are associated with bone mineral density (BMD) (Morrison et al., 1994). In recent years, with the development of microarray and next-generation sequencing technology, genome-wide association studies (GWAS) have been considered as powerful tools to investigate the genetic architecture of complex diseases (Liu G. et al., 2018). Especially since 2007, the GWAS have identified hundreds of susceptibility loci for osteoporosis and BMD (Estrada et al., 2012; Richards et al., 2012; Kemp et al., 2017; Morris et al., 2019). However, the vast majority (>80%) of reported genome-wide significant susceptibility loci are located in non-coding regions of the genome and provide limited information about the genetic mechanisms of osteoporosis (Liu G. et al., 2019; Manolio et al., 2009).

To clarify the complexities of osteoporosis genetic architecture in both coding and non-coding regions, we provided a comprehensive insight into the genetic and epigenetic mechanisms of osteoporosis and BMD based on the GWAS susceptibility loci. We delineated the genetic architecture of osteoporosis and BMD, and estimated their genetic correlation. Then, we identified the pathways and epigenetic regulatory elements that may be involved in the pathogenesis of osteoporosis and BMD. Finally, we further integrated GWAS and eQTL data to identify the potential functional target genes of the osteoporosis susceptibility loci.

## Materials and Methods

### GWAS Summary Datasets

The GWAS summary data of BMD and osteoporosis were downloaded from the UK Biobank. The BMD dataset includes 206,496 individuals and the osteoporosis dataset includes 35,736 patients and 355,405 controls.

### Mapping of SNPs to RefSNP ID and Gene

The human SNP (dpSNP147) and gene (GRCh37) position data were downloaded from NCBI. All the SNPs were mapped to the corresponding RefSNP ID and gene symbol (located within 10 kb upstream or downstream of the SNP) based on the position information (Wang et al., 2010).

### Estimation of Genetic Correlation

SNP-based genetic correlation between osteoporosis and BMD was calculated using LD Score regression (LDSC) (Yang et al., 2011). The regression was performed using pre-computed LD scores based on 1000 Genomes European data. To prevent the bias from the variable quality, we removed the variants that are InDels, not in 1000 Genomes European data, strand ambiguous SNPs, SNPs with duplicated rs numbers and SNPs with minor allele frequency (MAF) <0.01.

### Pathway Enrichment Analysis

bone mineral density and osteoporosis susceptibility genes that contain at least one significant susceptibility locus were identified (*P* < 5.00E-08), and the KEGG pathway and GO term enrichment analysis were performed using the CPDB database (Kamburov et al., 2013) based on the BMD and osteoporosis susceptibility genes, respectively. In this study, the adjusted *P*-value threshold for enrichment analysis is 0.05.

### Regulatory Element Enrichment Analysis

Regulatory element enrichment analysis was performed using various regulatory data from the ENCODE and Roadmap Epigenomics projects with GARFIELD software (Iotchkova et al., 2016; Cheng et al., 2018). The fold enrichment (FE) was calculated at different GWAS *P*-value thresholds (0.1 to 1E-08) after removing the known confounders such as local linkage disequilibrium, local gene density, matched genotyping variants and MAF (Iotchkova et al., 2016). The regulatory element enrichments were tested for various regulatory elements including open chromatin regions, DHSs, transcription factor binding sites and different types of epigenomic markers.

### Summary-Data-Based Mendelian Randomization Analysis

We applied a summary-data-based Mendelian randomization (SMR) method integrating osteoporosis and BMD GWAS summary-level data with expression quantitative trait locus (eQTL) data to identify target genes regulated by osteoporosis and BMD susceptibility loci (Zhu et al., 2016). SMR is an instrumental variable analysis approach that uses genetic SNP as an instrumental variable (*Z*) to test whether the effect of SNP (*Z*) on the outcome (*Y*) is mediated by gene expression (*X*) (Pavlides et al., 2016). The estimation of the effect size of *X* on *Y* (β_*XY*_) can be expressed as β_*XY*_ =β_*zy*_/β_*zx*_, where β_*zy*_ is the effect size of *Z* on *Y* and β_*zx*_ is the effect size of *Z* on *X*. GTEx blood eQTL data were used in the SMR analysis (Jiang et al., 2014; GTEx Consortium, 2017), and only SNPs within 1 Mb of the transcription start site are included in this study.

## Results

### Estimation of Genetic Correlation Between BMD and Osteoporosis

bone mineral density and osteoporosis show a high degree of clinical correlation (Kemp et al., 2017). To investigate whether there is a genetic correlation between the two phenotypes, we analyzed the genetic architecture between BMD and osteoporosis. The BMD and osteoporosis GWAS summary-level data were downloaded from the UK Biobank (Bycroft et al., 2018). We applied a cross-trait LDSC method to estimate genetic correlation by looking for correlations in effect sizes of all SNPs between BMD and osteoporosis (Yang et al., 2011; Bulik-Sullivan et al., 2015).

**FIGURE 1:**
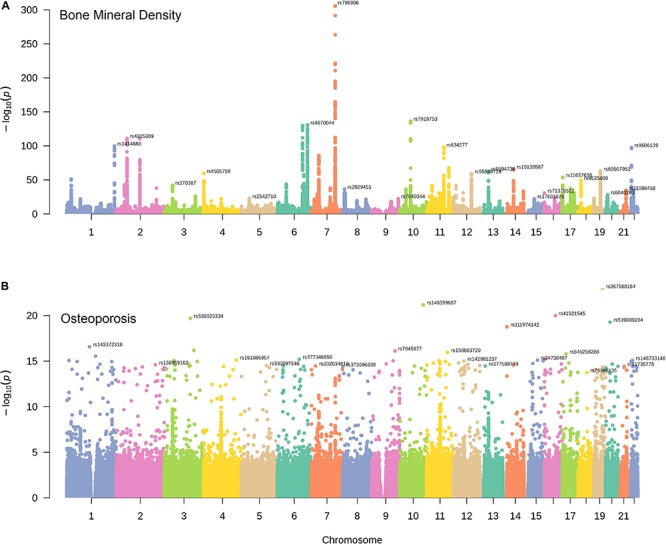
The distribution of BMD and osteoporosis susceptibility loci. Manhattan plots of single nucleotide variant associations for BMD **(A)** and osteoporosis **(B)** identified by UK Biobank. The top-SNP associations on each chromosome are annotated on the plot.

The chromosome distribution of BMD and osteoporosis susceptibility loci were displayed graphically in Figure 1. We obtained a statistically significant negative genetic correlation between BMD and osteoporosis (ρ*_*g*_* = −0.57, *P* = 6.32E-37). In other words, the genetic variants associated with increased risk of osteoporosis tend to induce a decreased BMD level, which conforms with the clinical pathology. It also has been proven that BMD susceptibility loci are significantly enriched for the clinically relevant therapeutic targets of osteoporosis (Richards et al., 2012; Nelson et al., 2015). In our results, BMD and osteoporosis were found to have many common susceptibility loci, 1578 of 5191 osteoporosis susceptibility loci were also associated with the level of BMD (*P*_*gwas*_ < 1.0E-04). The common susceptibility loci between BMD and osteoporosis can be found in Supplementary Table S1 and Supplementary Figure S1.

### Pathway Enrichment Analysis of BMD and Osteoporosis Susceptibility Loci Located in Protein-Coding Regions

To better understand the potential biological characteristics of the BMD and osteoporosis susceptibility loci located in the protein-coding regions, we mapped all the BMD and osteoporosis susceptibility loci to their corresponding genes based on the position information and conducted a pathway enrichment analysis of all mapped BMD and osteoporosis susceptibility genes with CPDB database. The susceptibility genes of BMD are significantly enriched in 17 KEGG pathways and 58 GO terms, osteoporosis susceptibility genes are enriched in 2 GO terms. More detailed results about all significant pathways can be found in Supplementary Table S2.

**FIGURE 2:**
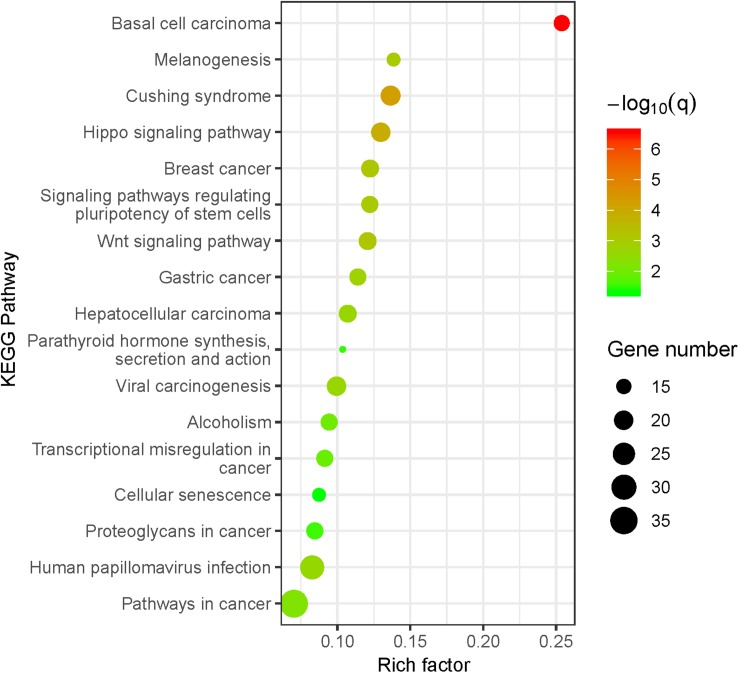
KEGG pathway enrichment analysis for BMD susceptibility loci located in protein-coding regions. The size of the point means the gene number both in BMD susceptibility genes and KEGG pathways. The color of point means enrichment significance (–log_10_ *Q*-value). The pathways were sorted by the rich factor (the ratio of BMD susceptibility gene number in this pathway to gene number in this pathway term).

Our results indicated that the BMD susceptibility genes are significantly enriched in bone-related biological pathways (Figure 2). The Wnt/β-catenin and Hippo signaling pathways have been found to play crucial roles in the osteoclasts and osteoblasts balancing (Liu Y. et al., 2019; Saad, 2019), and the GO enrichment results further supported the findings that BMD susceptibility genes are significantly enriched in biological processes of ossification (*P* = 3.86E-09). What’s more, both the glucocorticoid (Cushing syndrome) and parathyroid hormone have profound effects on the homeostasis of bone through multiple cellular and molecular mechanisms (Toth and Grossman, 2013; Wein and Kronenberg, 2018). Besides, we also found that many enriched KEGG pathways for BMD are associated with various types of cancer. It has been reported that cancer is one of the risk factors for osteoporosis due to the effects of cancer cells and cancer-specific therapies on bone cells (Drake, 2013). Therefore, the mutations located in BMD-related susceptibility genes play important roles in the osteoporosis pathogenesis by the dysregulation of the osteoblastic and osteoclastic biological processes.

### Regulatory Element Enrichment Analysis of BMD and Osteoporosis Susceptibility Loci Located in Non-coding Regions

The great majority of significant susceptibility loci identified by GWAS are located in non-coding regions such as regulatory elements (Medina-Gomez et al., 2018). In order to investigate whether the susceptibility loci of osteoporosis and BMD are significantly enriched in genomic regulatory elements, we performed a regulatory element enrichment analysis with LD correction (Iotchkova et al., 2016) to calculate FE values for regulatory elements at various genome-wide significant thresholds using regulatory data obtained from the ENCODE and Roadmap Epigenomics project (Supplementary Table S3).

We found that both the BMD and osteoporosis susceptibility loci (*P* < 1.00E-03) are significantly enriched in 5′ UTR region (OR_*BMD*_ = 3.48, *P*_*BMD*_ = 2.6E-02; OR_*OP*_ = 2.95, *P*_*OP*_ = 2.7E-03) according to the physical locations. The 5′ UTR region plays a regulatory role in RNA translation because it contains multiple functional elements (Araujo et al., 2012). The previous pathologic studies found that SNPs in 5′ UTR region can lead to abnormal expression of osteoporosis-related genes, including *TNFRSF11B* and *CYP17* (Tofteng et al., 2004; Krela-Kazmierczak et al., 2016). Our studies provide further evidence that a portion of susceptibility loci may increase osteoporosis risk by 5′ UTR-mediated regulation of BMD-related genes.

**FIGURE 3:**
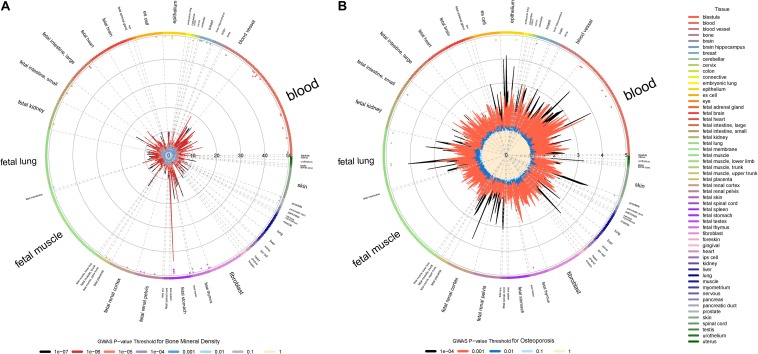
Regulatory element enrichment analysis of BMD and osteoporosis susceptibility loci located in non-coding regions. **(A)** Bone mineral density. **(B)** Osteoporosis. Radial lines with different color show FE values at their corresponding GWAS *P*-value thresholds for all ENCODE and Roadmap Epigenomics DNase I hypersensitive cell lines, sorted by tissue on the outer circle. The font size of tissue is directly proportional to the number of cell lines. The significance of enrichment for a given cell line is marked by the dots in the inner ring of the outer circle.

We also found that BMD and osteoporosis susceptibility loci (*P* < 1.00E-04) are significantly enriched in DHSs across different blood cells, especially in various subtypes of leukemia and normal blood lymphocyte (Figure 3). DHSs cover many kinds of gene regulatory elements such as enhancers, silencers and locus control regions. It has been proven that there are many osteoclast-specific DHSs located in well-known osteoclast transcription factor binding sites during early osteoclastogenesis (Inoue and Imai, 2014). What’s more, both the immune system and leukemia have significant effects on pathogenic mechanisms of osteoporosis (Cagnetta and Patella, 2012; Frisch et al., 2012). The dysregulation of immune cells may directly or indirectly modulate bone metabolism and remodeling through the secretion of various proinflammatory cytokines (Faienza et al., 2013; Srivastava et al., 2018). A significant decrease of BMD was also observed in patients with leukemia (Massenkeil et al., 2001), and the leukemic cells can also influence bone health through altering osteoblastic and osteoclastic functions (Frisch et al., 2012). Therefore, our results of regulatory element enrichment analysis indicated that the BMD and osteoporosis susceptibility loci are significantly enriched in DHSs of peripheral blood immune cells, which may induce bone microarchitectural deterioration by the dysregulation of peripheral blood immune cells.

### Identification of Target Genes of BMD and Osteoporosis Susceptibility Loci by Integrating GWAS and eQTL Data

bone mineral density and osteoporosis susceptibility loci were found to be significantly enriched in 5′ UTR and DHSs. To identify the potential functional target genes of the susceptibility loci of osteoporosis, we performed a SMR analysis by integrating the GWAS data with eQTL summary data (Wu et al., 2018). The blood eQTL summary data were obtained from the GTEx project (GTEx Consortium, 2017). We mapped all the susceptibility loci to GTEx eQTL target genes in cis-regions and then linked them to BMD and osteoporosis.

**FIGURE 4:**
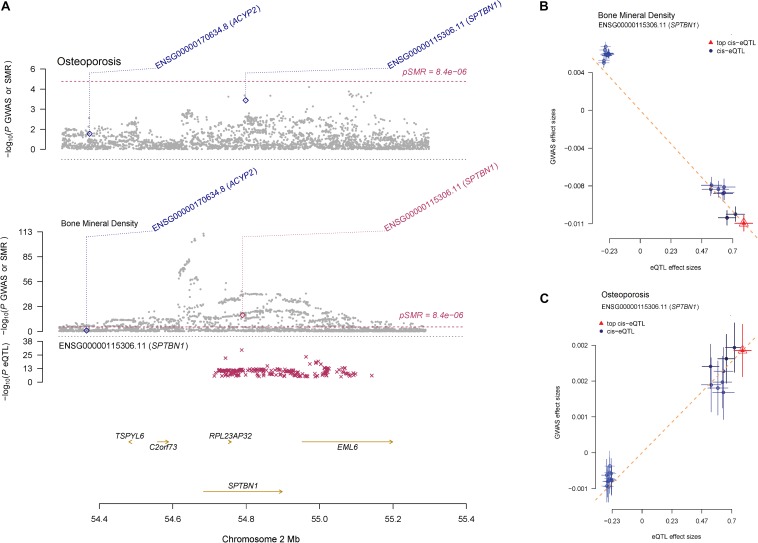
The regulation mechanism of *SPTBN1* locus for BMD and osteoporosis. **(A)** *P*-values of GWAS (gray dots) for BMD (top) and osteoporosis (middle) and *P*-value for the SMR test (diamonds) using the GTEx blood eQTL data. The bottom plot shows the eQTL *P*-values from GTEx blood tissue for the *SPTBN1* gene (red stars). The dots shown in the plot include all the SNPs at these loci in the GWAS and eQTL summary data, respectively. **(B, C)** Effect sizes of SNPs from BMD **(B)** and osteoporosis **(C)** GWAS data against those from the GTEx blood eQTL data. The orange dashed lines represent effect size (*b*_*xy*_) of eQTL on phenotype. Error bars are the standard errors of SNP effects.

Summary-data-based Mendelian randomization identified that 50 target genes were associated with BMD (*P*_*SMR*_ < 8.4E-06) (Supplementary Table S4), 15 of the 50 target genes were also associated with osteoporosis (*P*_*SMR*_ < 0.05), suggesting that the expressions of those genes may have a connection with both BMD and osteoporosis. A notable example is the SNPs located in the *SPTBN1* coding region, where the SNP-association signals are significant and consistent across GWAS and eQTL datasets with *P*_*SMR*_ = 3.15E-19 for BMD and *P*_*SMR*_ = 2.20E-04 for osteoporosis (Figure 4A). The effect sizes of SNPs show the overexpression of SPTBN1 associated with decreased BMD level (Figure 4B) and increased osteoporosis risk (Figure 4C). The regulation of *SPTBN1* showed a possible pathogenic mechanism for both BMD and osteoporosis.

Among the 15 target genes of BMD and osteoporosis susceptibility loci, *SPTBN1* is a molecular scaffolding protein which has been recognized as an osteoporosis susceptibility gene (Estrada et al., 2012; Chen et al., 2016), and it shows co-expression with alpha-actinin binding and cell adhesion gene in the bone (Calabrese et al., 2017). The overexpression of *ASB16-AS1* also can increase the expression of osteoblastogenesis related genes (Meng et al., 2018). The *SUPT3H-RUNX2* locus associated with bone-related phenotypes including height (Lango Allen et al., 2010) and BMD (Estrada et al., 2012) through regulation of osteoblastic differentiation and skeletogenesis (Rice et al., 2018). Above all, our integration research of GWAS and eQTL data identified 15 target genes of both BMD and osteoporosis susceptibility loci, and the expression of those genes may play important roles in osteoblastic biological processes.

## Discussion

In this study, we provided comprehensive insights into the genetics and epigenetic mechanisms of osteoporosis and BMD based on the GWAS summary data. The genetic architecture demonstrated that BMD and osteoporosis share many common susceptibility loci, which allowed us to elucidate pathogenic mechanisms of osteoporosis by integrating the BMD and osteoporosis GWAS summary data. Further, the pathway and regulatory element enrichment analysis found that the susceptibility loci located in both coding and non-coding regions play crucial roles in determining BMD and the pathophysiology of osteoporosis.

The pathway analysis demonstrated that both cancer, Cushing syndrome, Parathyroid hormone, Wnt/β-catenin and Hippo signaling pathways may play crucial roles in osteoporosis pathogenesis by the dysregulation of the osteoblastic and osteoclastic biological processes. What’s more, the regulatory element enrichment analysis found BMD and osteoporosis susceptibility loci are more often located in 5′ UTR and DHSs of peripheral blood immune cells. Finally, we found the expression of 15 target genes (*SPTBN1, ASB16-AS1, SUPT3H-RUNX2*, etc.) regulated by susceptibility loci, are associated with BMD and osteoporosis by integrating GWAS summary data with eQTL data.

In summary, we integrated GWAS susceptibility loci with multi-omics datasets to uncover the molecular mechanisms and identify target genes of osteoporosis-associated variants. Our findings shed light on the genetics and epigenetic mechanisms of osteoporosis and provided new clues for effective target therapeutics of osteoporosis. In the future, further researches about the function of those target genes are promising to gain a better understanding of osteoporosis.

## Data Availability Statement

Publicly available datasets were analyzed in this study. This data can be found here: The GWAS summary data can be downloaded from UK Biobank (http://www.nealelab.is/uk-biobank) under the phenotype code 3148 (bone mineral density) and 20002_1309 (osteoporosis).

## Author Contributions

HL designed the experiments. WZ obtained the data from UK Biobank. WZ, PW, HD, EZ, XY, SC, TF, LJ, GM, and CC analyzed the data. WZ and HL wrote the manuscript. All authors read and approved the manuscript.

## Conflict of Interest

The authors declare that the research was conducted in the absence of any commercial or financial relationships that could be construed as a potential conflict of interest.

## Supplementary Material

FIGURE S1 The common susceptibility loci between BMD and osteoporosis.

TABLE S1 The shared genetic susceptibility loci between osteoporosis (OP) and bone mineral density (BMD).

TABLE S2 **(A)** The results of KEGG pathways enrichment analysis for BMD susceptibility genes. **(B)** The results of GO pathways enrichment analysis for BMD susceptibility genes. **(C)** The results of GO pathways enrichment analysis for Osteoporosis susceptibility genes.

TABLE S3 **(A)** The results of regulatory element enrichment analysis for BMD susceptibility loci. **(B)** The results of regulatory element enrichment analysis for Osteoporosis susceptibility loci.

TABLE S4 The results of summary-data-based Mendelian randomization analysis.
